# Assessing individual physiological variability and future performance phenotypes is essential for predicting the resilience of fish populations to anthropogenic climate change

**DOI:** 10.1093/conphys/coaf043

**Published:** 2025-06-24

**Authors:** Lauren A Bailey, Amber Robyn Childs, Nicola C James, Murray I Duncan, Brett A Pringle, Warren M Potts

**Affiliations:** Department of Ichthyology and Fisheries Science, Prince Albert Street, Makhanda, Rhodes University, Grahamstown 6139, South Africa; Department of Ichthyology and Fisheries Science, Prince Albert Street, Makhanda, Rhodes University, Grahamstown 6139, South Africa; Department of Ichthyology and Fisheries Science, Prince Albert Street, Makhanda, Rhodes University, Grahamstown 6139, South Africa; South African Institute for Aquatic Biodiversity, Private Bag 1015, Grahamstown 6140, South Africa; Department of Ichthyology and Fisheries Science, Prince Albert Street, Makhanda, Rhodes University, Grahamstown 6139, South Africa; Department of Ichthyology and Fisheries Science, Prince Albert Street, Makhanda, Rhodes University, Grahamstown 6139, South Africa; Department of Environment, University of Seychelles, Anse Royale, Mahe, Seychelles; Blue Economy Research Institute, University of Seychelles, Anse Royale, Mahe, Seychelles; Department of Ichthyology and Fisheries Science, Prince Albert Street, Makhanda, Rhodes University, Grahamstown 6139, South Africa; South African Institute for Aquatic Biodiversity, Private Bag 1015, Grahamstown 6140, South Africa

**Keywords:** Aerobic scope, physiological performance, physiological phenotypes, physiology, thermal variability

## Abstract

Changes in ocean temperature are expected to have a considerable effect on fishes through the impact of temperature on physiological performance, vital energetic processes (i.e. metabolism, foraging and swimming style) and reproductive fitness. To understand the sensitivity of an exploited population of *Chrysoblephus laticeps* in to temperature variability, intermittent-flow respirometry was used to quantify and compare changes in metabolic rate and aerobic scope under different temperatures (10, 16, 21 and 24°C) mimicking thermal variations experienced in the home range of this species. A total performance score was developed to represent aerobic performance across the range of test temperatures. This score was calculated for each temperature from the lower (25%), mid (50%) and upper (75%) percentiles of the aerobic scope range available for the species. The results of this study identified heterogeneity in physiological performance phenotypes amongst individuals of the exploited population. There was significant variation in the aerobic performance of high, intermediate and low performers at higher temperatures. However, differences in performance were not significant at low temperatures, where several intermediate performers maintained high performance. High performers maintained high rates of physiological performance across a broad range of temperatures, whereas low performers were physiologically limited outside of their optimal thermal range. These results suggest that individuals with a broad aerobic scope (i.e. high aerobic scope (AS) values across a range of temperatures) may likely be the most resilient to short-term thermal variability caused by marine heat waves and upwelling events in temperate coastal environments. Since the shape of thermal performance curves differs between individuals and reflects the range at which individuals can function above specified performance thresholds, individual thermal performance must be measured repeatedly in the same individual over a thermal gradient. An understanding of physiological phenotypic diversity amongst individuals is critical to understand the impacts of thermal variability on fished populations.

## Introduction

Fish populations are currently experiencing the consequences associated with a rapidly changing climate (Bates *et al*., 2018; Bates *et al*., 2019). Besides directional changes in mean environmental conditions, the global increases in mean sea surface temperatures have altered wind regimes (Rhein *et al*., 2013), driving short-term shifts in local ocean weather (Bates *et al*., 2018; Bates *et al*., 2019). The consequences of these changing means and rapid fluctuations in environmental conditions for marine fishes will be determined by their physiological tolerances. Fish may alter their tolerance range by shifting their physiological performance curves either by broadening their tolerance to a range of stressors, maximizing their performance at extremes (Pörtner and Knust, 2007; Chown *et al*., 2010), or altering their behaviour to seek out refugia from external stressors (Wong and Candolin, 2015).

As fish are ectotherms and their body temperatures track ambient thermal conditions (Rijnsdorp *et al*., 2009; Pauly, 2010), changes in these thermal conditions are one of the greatest threats to fish (Rhein *et al*., 2013; Potts *et al*., 2015). From a physiological perspective, changing temperatures will result in alterations to the rates at which fish will oxidize substrates to produce energy. This will impact both vital processes (such as foraging and swimming style; Brownscombe *et al*., 2014; Johansen *et al*., 2014), the energy remaining for reproductive fitness and their demography and distribution (Rijnsdorp *et al*., 2009).

The concept of oxygen-and-capacity-limited thermal tolerance (OCLTT) is thought to be useful for assessing the influence of temperature, amongst other environmental factors, on the performance and abundance of ectotherms (Pörtner and Knust, 2007; Pörtner *et al*., 2018)*.* The OCLTT concept addresses the thermal constraints on the capacity for the supply of oxygen to the animal relative to its oxygen demand (at both the molecular and whole-animal level; Pörtner *et al*., 2018). The remaining aerobic energetic capacity contributes to an organism’s performance (i.e. locomotion, growth, reproduction) within the thermal limits of the organism. The measure of energetic capacity is often approximated through measurements of aerobic scope, defined as the difference between standard metabolic rate (SMR; the rate at which an animal oxidizes metabolic substrates to produce the energy required to maintain homeostasis in a post-absorptive, inactive state; McNab, 2002; Metcalfe *et al*., 2016) and maximal metabolic rate (MMR; maximal locomotor activity. Although the OCLTT concept has received much criticism (Clark *et al*., 2013; Pörtner *et al*., 2018), e.g. it has been argued that the OCLTT should not be applied to tropical species with steady increases in MMR that are unlikely to reach maximum performance in natural conditions (Chown *et al*., 2010; Pörtner *et al*., 2018; Neubauer and Andersen, 2019), the OCLTT is relevant to species that may reach their oxygen limits in their ecological reality. Examples include organisms living in highly variable temperate coastal environments with frequent short-term exposure to both extreme high and low temperatures (Bates *et al*., 2018, 2019; Pörtner *et al*., 2018). In these environments, the OCLTT can be used to assess short-term tolerance to extreme environmental changes (Pörtner *et al*., 2018).

In general, the future physiological responses of species to the impacts of a changing climate have been predicted based on the population mean aerobic capacity for thermal change (Bennett, 1987; Williams, 2008; Ward *et al*., 2016; Guscelli *et al*., 2019). This approach is problematic as it assumes that the response of all individuals of a species will be similar and that thermal adaptation will be slow (Bennett, 1987; Ward *et al*., 2016; Guscelli *et al*., 2019). However, it must be considered that there may be 2- to 3-fold variation in the physiological traits such as metabolic rates of between individuals and that these are partially heritable (Metcalfe *et al*., 2016; Taboun, 2020; Long *et al*., 2021). As such, some individuals may be able to perform at a broader range of thermal conditions and since their genes would selectively be passed on to the next generation, the rate of adaption of species may be considerably faster than previously thought (Metcalfe *et al*., 2016; Taboun, 2020; Long *et al*., 2021). Therefore, the determination of intrapopulation (i.e. individual-level) aerobic performance is critical for understanding which fishes will be more resilient to a rapidly changing climate (Bennett, 1987; Williams, 2008; Guscelli *et al*., 2019). In the context of changing temperatures, this can be done by repeatedly measuring the aerobic performance of the same individual across a thermal gradient (Killen *et al*., 2021). This information can then be used to classify individual metabolic phenotypes (high-performance phenotypes with a high or broad aerobic scope, vs low-performance phenotypes with a narrow aerobic scope, in thermally variable regions) and provide us with an understanding of how resilient the population may be to change by quantifying the proportion of high-performance phenotypes.

While individual-level physiological information is critical to assess phenotypic diversity of physiological traits, an understanding of the likely performance of different metabolic phenotypes in future conditions is also necessary. For example, species that are distributed in the tropics or polar regions, where the thermal environment is generally stable, will mostly comprise individuals that perform optimally at a narrow range of temperatures and this will be reflected by a steep performance curve (Chown *et al*., 2010; Neubauer and Andersen, 2019). The resilience of these fishes to warming conditions will largely depend on the proportion of individuals that either have broader thermal performance curves or perform optimally at higher temperatures. In contrast, the proportion of individuals with the broadest thermal performance curves will determine the resilience of a fish population in an increasingly variable temperate environment, where individuals generally have broader thermal performance curves (Pörtner and Knust, 2007; Pörtner, 2008; Clark *et al*., 2013). Thus, the prediction of the response of a species to rapid environmental change, requires not only an understanding of the individual physiological variability within species but also the relative abundance of physiological phenotypes that are likely to perform in future conditions (future physiological performance phenotypes). While aerobic scope (AS) may provide a suitable metric to test intraspecific variability in the thermal physiological traits of fishes, its utility for the classification of individual metabolic phenotypes (i.e. using a repeated measures approach) and identification of future high-performance phenotypes in exploited fishes has not been adequately explored.

The roman seabream *Chrysoblephus laticeps* (Valenciennes*, 1830*) is a reef-associated (up to <100 m depth) sparid fish that is endemic to southern Africa, with the species primarily distributed along the thermally variable warm-to-temperate southern coastline of South Africa. This slow-growing, highly resident reef fish is heavily exploited by both commercial and recreational ski-boat line fisheries in South Africa (Duncan *et al*., 2019). Social behavioural traits, exploitation (Kerwath *et al*., 2007), hardiness in captivity (Duncan *et al*., 2019) and existing information on the effect of climate change (temperature and acidification) on the metabolism of both the adult (Duncan *et al.,* 2019) and larval population make this a model species to address research questions on how individual physiological traits influence resilience to thermal variability. Duncan *et al.* (2019) did not identify a statistically significant decline in population-level performance at acute warming temperatures of 24°C, likely due to a low sample size for the exploited population and different experimental design (i.e. the lack of repeatable measures of performance in the same individual prevented the detection of the full thermal performance range of each individual; Killen *et al*., 2021). However, this temperature was found to be close to the maximum cardiac breakpoints for *C. laticeps* (i.e. 23.92 and 25.19°C; Skeeles *et al.*, 2020). The southern coast of South Africa is experiencing an increase in thermal variability with an increase in the intensity and frequency of both marine heat waves (Schlegel *et al*., 2017) and upwelling events (Duncan *et al*., 2019). Duncan *et al*. (2019) found that extreme cold events cause a cold shock in red roman. The increased thermal variability along the southern coast of South Africa, as a result of upwelling and marine heat wave events, will require fishes to have a broad thermal performance curve (as such, a broad aerobic scope phenotype is likely to be selected as the future high-performance metabolic phenotype that will best cope with increases in thermal variability).

The aim of this manuscript is to assess the metabolic performance of individuals from an exploited population of *C. laticeps* in order to 1) examine phenotypic variability within the population 2) categorize individuals as high or low performers and 3) identify the proportion of individuals that are likely to maintain their aerobic performance in the increasingly variable future conditions. To do this, we repeatedly measured the aerobic scope of the same individual across a thermal gradient currently experienced by these fish in the wild. We used a percentile method to assess individual thermal performance. We developed a scoring system to categorize individuals into low-, medium- and high-performance phenotypes and theorized that the high- performance phenotypes will be most competitive in the more variable future thermal environment. It is suggested that this framework can be used to assess the likely physiological performance and resilience of fish populations (and species) to a rapidly changing climate.

## Materials and Methods

### Study site

**Figure 1:**
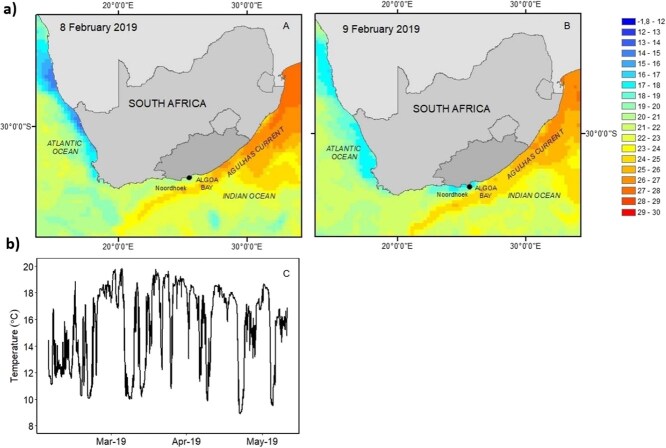
Mean sea surface temperatures at Noordhoek collected on 8 and 9 February 2019 (a) at ±10–20 μm depth in Celsius (°C) using the Group for High Resolution Sea Surface Temperature (GHRSST; Level 4 Multiscale Ultrahigh Resolution L4). Figure 1b is the mean daily *in situ* data collected at 14 m depth at Noordhoek during March 2019 (as per Skeeles 2022). The GHRSST for sea surface temperature makes use of interpolated wavelength functions on a worldwide 0.011 degree grid. Sea surface temperature data was obtained from the NASA Advanced Microwave Scanning Radiometer-EOS, the NASA Aqua and Terra platforms, Moderate Resolution Imaging Spectroradiometer (MODIS) and the US Navy Windsat microwave radiometer. *In situ* sea surface temperature observation data was obtained from the NOAA iQuam project.

The area offshore of Noordhoek (33°58’S 25°38′E), Gqeberha, which extends along an exposed Cape headland west of Algoa Bay, falls within a dynamic upwelling region along the southern South African coastline (Fig. 1). The exposed headland experiences greater sea temperature variability than Algoa Bay (Duncan *et al*., 2019). For example, temperatures in Noordhoek can drop from 22 to 11°C (measured at 14 m depth) over a matter of hours following an upwelling event (Skeeles *et al.,* 2020). These cold temperatures may remain for a 1- to 4-day period. Changes in the El Nino Southern Oscillation (ENSO) are likely to result in more intense and frequent wind-driven upwelling events (Duncan *et al*. 2019), particularly along prominent coastal headlands.

### Fish capture from study site and fish husbandry:

To measure individual aerobic scope performance to different temperatures, 44 *C. laticeps* individuals were captured using hook and line at depths of between 12 and 25 m off a small vessel launched from Noordhoek in November 2019 and April and June 2021.

Captured specimens were immediately vented with a hypodermic needle and transferred to a 1000-l tank containing fresh seawater. On shore, fish were moved to a circular holding tank (1000 l), which was supplied with fresh seawater at a rate of 225 l/min via a submersible pump. Following a 5-hour holding time, all specimens in the 1000-l tank received a continuous supply of pure oxygen for transport to the laboratory aquaculture facilities in the NRF-SAIAB Aquatic Ecophysiology Research Platform at the Department of Ichthyology and Fisheries Science, Rhodes University.

Fish were placed into two 5900-l cylindrical holding tanks and maintained for a 2-week acclimation period at a constant temperature of 16°C (controlled through a 1.5 kW titanium hot rod submerged heating element, wall-mounted air conditioner and STC-1000 temperature controllers) and a light cycle of 9.5 L:14.5 D. Control temperatures of 16°C are considered optimal for the species and had no effect on performance classification. Holding tanks were connected to a filtration system made up of a 750-l slimline sump, protein skimmer (UltraZap with submerged Jebao DCP5000 pump), bubble bead filter (BBF-200-COMP, Wilpet Koi Products), fluidized bed biological filter (750-l slimline tank with SuperActiFlo Media) and UV sterilizer (UV 55 W, UltraZap Pro UVS-55). A pool pump (Speck Porpoise 0.75 kW) was used to recirculate water through the system. Air was supplied to the holding tanks and bed filter via a 2.2 kW blower outside the holding room*.* Daily measurements of O_2_ and pH were done using an Aqualytic pH meter, salinity was measured using a refractometer and ammonia and nitrite were measured using Salifert test kits. Fish were fed a maintenance diet of squid (*Loligo reynaudii*) every second day. Fish were tagged using colour combinations of Visible Elastomer Implant (VIE) tags (Northwest Marine Technology, USA) via intramuscular injection below the dorsal fin for the purpose of individual identification. Fish were handled according to Rhodes University ethics guidelines and approval (2019-0991-3182).

### Respirometry:

Metabolic rate measurements were conducted in an experimental room that housed a 2100-l cylindrical tank connected to a filtration system comprised of a protein skimmer, bubble bead filter (BBF-100 COMP), fluidized bed filter and UV sterilizer (UV 55 W, UltraZap Pro UVS-55). Oxygen was maintained at 100% saturation in the cylindrical tank and fluidized bed filter via air supply from a 2.2 kW blower. Water was pumped from the cylindrical tank into four glass tanks containing respirometers.

The respirometry chambers (29.72 l; 45 cm length; 29 cm internal diameter) were small enough to measure oxygen consumption by individual fish within a limited time span, but of sufficient size to prevent rapid oxygen depletion. Respirometers were made from thick-walled Perspex and had a g:ml ratio between 20 and 70 based on fish size, to comfortably house the specimen while limiting movement within the chamber. Openings with silicon O-ring seals were bolted closed. An internal pump (880 l/h 15 W SOBO pump) mounted onto the rear of the respirometer, along with an oxygen-impermeable PVC recirculation loop, was used to continuously mix water within the chambers to distribute oxygen and prevent stratification. Water from the recirculation loop was pumped via a peristaltic pump (Ismatek IPC-N-24 precision cassette pump with 24 multi-channel pump head) through a cell with an optical oxygen sensor (OXFTC, Pyro Science GmbH), from which oxygen concentrations (mg.l^−1^) were taken using a Firesting oxygen reader (FSO2-4, Pyro Science GmbH; and bare optical fibres SPFIB-BARE, Pyro Science GmbH). Pyro Oxygen Logger Software (Pyro Science GmbH) was used to record oxygen concentrations. One-way check valves on the flush pump and overflow lines prevented oxygen leaks. Following the closed measurement period, the flow of fresh oxygenated seawater into each of the four respirators was controlled by a flush pump (Bubble Magus DC 10000 s guaranteeing a minimum 4-fold flush volume ratio; controlled with a digital timer Eliro VODDTS), eliminating waste products.

Intermittent-flow respirometery was used, as per the protocol of Clark *et al*. (2013), to determine the oxygen consumption rate of the 44 *C. laticeps* individuals. Fish were fasted for 36 h prior to respirometry measurements to evacuate their guts and decrease post-feeding increments in metabolic rate (Cutts *et al*., 2001; Clark *et al*., 2013). Each individual was exposed to three temperature treatments (a low of 10°C, an acclimation temperature of 16°C and a high of 21°C) 1 week apart. The order of the extreme temperature treatments was randomly assigned amongst individuals, i.e. half of the individuals began with the cold treatment, whereas the other half began with the warm treatment. The oxygen consumption (O_2_ per minute) of each individual was measured at low (10°C), acclimation (16°C) and high (21 or 24°C) temperatures. The low temperature and the high temperature respectively represented cold upwelling events and marine heat waves occurring in the home range of the species (Fig. 1). Initially, the high temperature treatment was 24°C. However, upon observing sub-lethal effects (in the first 16 individuals after being exposed to the high temperature), including delayed mortalities, it was decided to decrease the highest temperature treatment to 21°C for the remaining 28 individuals to ensure that they were not compromised for the remaining test temperatures.

To estimate the minimum energy required for maintenance, SMR was measured. Here, each individual was placed into each of the four respirometers for a 12-h acclimation period maintained at 16°C. Following this, temperature was either maintained at 16°C or increased or decreased by one degree every hour using an Aquaheat SF2020P heat pump, to mimic intense upwelling and marine heat wave events, until the low (10°C) and high (21 or 24°C) test temperatures were reached. Half of the sampled population was first exposed to 10°C, while the other half was first exposed to 21°C to control for experimental temperature effects. Twelve fish were exposed to the 24°C treatment instead of 21°C. Once the temperature had stabilized, oxygen consumption was recorded for 5 min, with flushing for 15 min for 24 h. A 24-h period was considered necessary for the measurement of SMR as it accounted for circadian rhythm changes in metabolic rate (Clark *et al*., 2013). To account for an increase in metabolic rate at high test temperatures (21 or 24°C), measurement periods were adjusted to 3 min, followed by 17-min flush periods.

**Table 1:** Classification of aerobic performance phenotypes

	**Low performer: <25% percentile (O**_**2**_ **.min** ^**−1**^**.kg** ^**−1**^**)**	**Intermediate performer: <50% percentile (O**_**2**_ **.min** ^**−1**^**.kg** ^**−1**^**)**	**Intermediate performer: >50% percentile (O**_**2**_ **.min** ^**−1**^**.kg** ^**−1**^**)**	**High performer >75% percentile (O**_**2**_ **.min** ^**−1**^**.kg** ^**−1**^**)**
*Points per temperature treatment*	*1 point*	*2 points*	*3 points*	*4 points*
*16°C*	<1.75	<2.6	>2.6	6 > 3.45
*10°C*	<2.59	<3.68	>3.68	>4.77
*21–24°C*	<3.35	<4.2	>4.2	>5.05
Total rank scores (between 3 and 12) based on the percentile cut-off, where ≤5 = low performer and ≥10 = high performer	**Total score ≤3–5:** **Low performer** Low performance across all temperatures (i.e. total score of 3). **OR** intermediate aerobic performance **ONLY** at optimal temperature (i.e. score of 3 at optimal temperature, and then LP scores of 1 at each of the other two temperatures, resulting in a total score of 5).	**Total score 6–9:** **Intermediate performer** High aerobic performance at optimal temperature and/or high **OR** low temperature		**Score** ≥**10–12:** **High performer** High aerobic performance at optimal, low **AND** high temperature

For maximum metabolic measurements (MMR), individuals were subsequently transferred to the 2100-l cylindrical tank where they were chased for 10 min until exhausted (or unresponsive to touching of the caudal fin; Clark *et al*., 2013). This was followed by 30 s of air exposure to ensure that the fish were completely exhausted and likely to reach the maximum O_2_ consumption rate during the recovery period (Clark *et al*., 2013). Individuals were then returned to respective respirometers for several hours (±5 h), until oxygen use stabilized near SMR measurement levels (Clark *et al*., 2013). After the termination of the experiment, the oxygen concentration in empty respirometers was measured for 3 h to record background respiration rates (as per Duncan *et al*., 2019).

Measurement and flushing periods during respirometry experiments were transformed into a dataset of several independent rates of oxygen consumption *(R*O_2_) for each individual (with a quality threshold of R^2^ > 0.9 to filter linear oxygen declines within measurement periods Duncan, 2018). The rate of oxygen consumption (mg.kg^−1^.h^−1^) for each measurement period (where the first minute of measurement was excluded) was calculated using Svendsen *et al*.’s (2016) equation (1):


(1) \begin{align*}\notag{RO}_2&=\left(\left(\frac{V_{re-}M}{W}\right)\left(\frac{\Delta \left[{O}_{2a}\right]}{\Delta t}\times 60\right)\right)\\&\quad-\left(\left(\frac{V_{re-}M}{W}\right)\left(\frac{\Delta \left[{O}_{2b}\right]}{\Delta t}\times 60\right)\left(\frac{V_{re}}{V_{re}-M}\right)\right) \end{align*}

where V_re_ = total volume of respirometer in litres, M = mass of individual in kilogrammes expressed in litres, W = mass of individual in kilogrammes, $\frac{\Delta \left[{O}_{2a}\right]}{\Delta t}$= slope of linear decrease in O_2_ concentration within measurement period, $\frac{\Delta \left[{O}_{2b}\right]}{\Delta t}$ = slope of linear decrease in O_2_ concentration in respirometer without specimen.

The SMR was calculated from the quantile of the lowest 20% of the *R*O_2_ data for each test temperature. Maximum metabolic rate, which increased after exhaustive exercise until O_2_ consumption rates reached a plateau, was recorded as the single highest *R*O_2_ measurement. Prior to accounting for allometric mass effects, the SMR and MMR *R*O_2_ data were corrected for temperature as a function of the Boltzmann factor (Brown *et al*., 2004; using the average activation energy of ectotherms; Gillooly *et al*., 2001). Rates of oxygen consumption were temperature corrected (*R*O_2(temp corrected)_) using the equation (2):


$$ {RO}_{2\left( temp\ corrected\right)}={RO}_2\times{e}^{\frac{-E}{kT}} $$

where E = average activation energy of ectotherms (0.63 eV; Gillooly *et al*., 2001), k = Boltzmann constant 8.617333 × 10^−5^ eV.K^−1^, T = absolute temperature in kelvin.

The allometric exponent (α) of mass scaling effects was then estimated through the slope of the linear regression between the natural logarithm of *R*O_2(temp corrected)_ and the natural logarithm of mass. The allometric exponent (α) of mass scaling effects was used to correct the data using the equation (3):


$$ {MO}_2=\frac{RO_2}{M^a} $$

where ${MO}_2$ = mass-corrected SMR or MMR and ${RO}_2$ = oxygen consumption rate for SMR or MMR$.M$ = mass of individual and $a$ = allometric exponent of mass scaling.

The absolute AS for each individual was then calculated by subtracting mass-corrected SMR from mass-corrected MMR using the equation (4):


$$ AS=MMR-SMR. $$

### Classification of high, intermediate and low aerobic performance phenotypes:

Because of the thermally variable environment and predicted increase in variability, a total performance score was developed to represent aerobic performance across the range of test temperatures (according to the percentile method as per Rousselet *et al*., 2017) (Table 1). This score was calculated for each temperature from the lower (25th), mid (50th) and upper (75th) percentiles of the aerobic scope range available for the species from this and a previous study (Duncan *et al.,* 2019). For each temperature, each individual received a rank score based on the percentile that included its aerobic scope (a total score of <3–5 is classified as a low performer, 6–9 is as an intermediate performer and 10–12 as a high performer). Rank scores were totalled for all temperatures, where an individual could receive an absolute minimum score of 3 (i.e. a score of 1 for each temperature) and maximum score of 12 (i.e. a score of 4 for each temperature). These scores were used to classify individuals according to the percentile method as high performers (i.e. the >75th percentile with a total score >9.75, rounded off as 10), intermediate performers (25–75th percentile) and low performers (i.e. the <25th percentile with a total score <5.25, rounded off as 5). An individual that received a score of ≤5 was classified as a low performer (i.e. may have performed moderately well at optimal temperatures, but may be limited by aerobic performance in a thermally variable environment, i.e. at thermal extremes). An individual that received a score of ≥10 was classified as a high performer (i.e. exhibited a broad tolerance to a thermally variable environment).

### Statistical analysis

A linear mixed effects (*lme*) modelling approach was used to model the relationship between temperature, metabolic rate and phenotype. This was implemented using the *lme4* package (Bates *et al*., 2015) in R version 3.3.3 (R Core Team, 2017) to account for data homoscedasticity (model assumptions were also checked using diagnostic plots) and repeated measures (i.e. each individual was measured at each of the three test temperatures. The effect of temperature on metabolic rate was tested by modelling a second order polynomial relationship between metabolic data and temperature, including fish ID and temperature, to consider the variation of each individual. To account for the varying relationship between metabolic rate and temperature of each individual, temperature was included as a random slope in the random effects structure with individual as the random intercept (i.e. temp|ID). There was no significant difference in the aerobic scope estimates of individuals that were tested first at 10°C and those tested first at 21 or 24°C, and therefore all fish batches were pooled for individual analyses; Appendix Table A2. Aerobic performance group (i.e. as a factor for high performers, intermediate performers and low performers) was added as a fixed effect. Sex, batch (low or high temperature first) and weight class (see Table A1 in the Appendix) were not significant in predicting individual metabolic data in the initial model (*P* > 0.05, *t* = 1.434, *df* = 77) and these variables were then excluded from further model analyses (i.e. data was pooled between the sexes, batches and weight classes). The model output was assessed using the ‘Dredge’ function with libraries ‘mvtnorm’ and ‘MuMIn’ from the ‘CRAN’ package (R Core Team, 2017).

## Results

### Metabolic variability

**Figure 2:**
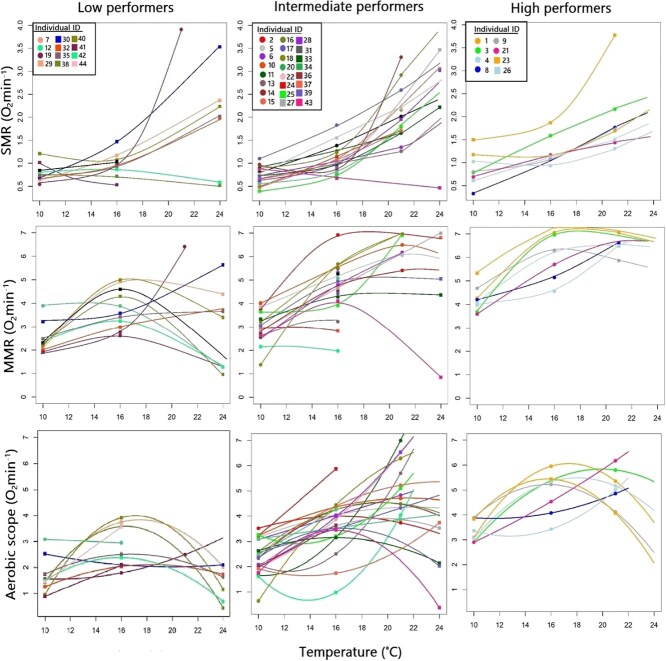
The change in standard metabolic rate, maximum metabolic rate and aerobic scope curves for high performers (>75% percentile), intermediate performers (25–75% percentile) and low performers (<25%) of an exploited *C. laticeps* population across a temperature gradient (10, 16, 21 and 24°C). An individual that was classified as a high performer exhibited a broad tolerance to a thermally variable environment. An individual that was classified as a low performer may have performed well at optimal temperatures, but may be limited by aerobic performance in a thermally variable environment.

**Figure 3:**
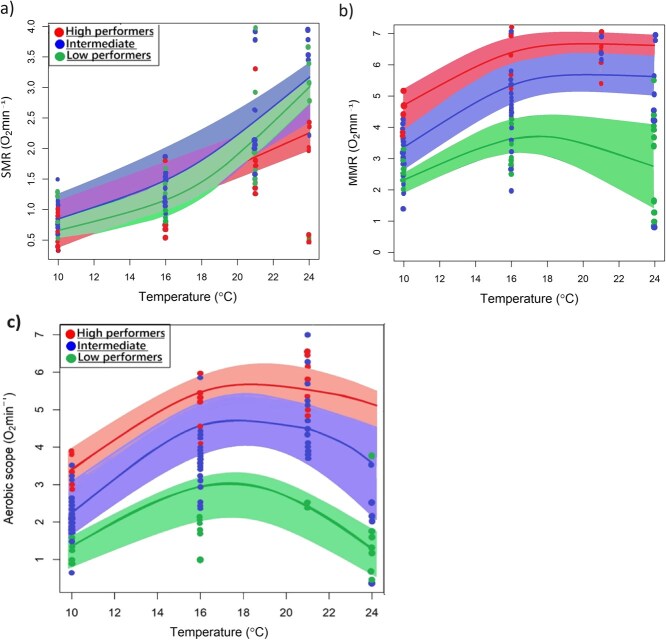
Overall standard metabolic (a), maximum metabolic rate (b) and aerobic scope curves (c) for high, intermediate and low performers for an exploited *C. laticeps* population across a temperature gradient (10, 16, 21 and 24°C) with shaded areas representing a 95% confidence interval. High performers exhibited a broad tolerance to a thermally variable environment. Low performers may have performed well at optimal temperatures, but may be limited by aerobic performance in a thermally variable environment.

Mass-corrected SMR varied amongst individuals and ranged from 0.465 to 3.748 O_2_.min^−1^ kg^−1^ across the various temperatures (Figs A1 and A2 in the Appendix, and Fig. 3a). At the three test temperatures, SMR ranged between 0.840 and 1.146 O_2_.min^−1^ kg^−1^ at 10°C, between 0.533 and 1.841 O_2_.min^−1^ kg^−1^ at 16°C, between 1.207 and 3.748 O_2_.min^−1^ kg^−1^ at 21°C and between 0.465 and 3.532 O_2_.min^−1^ kg^−1^ at 24°C (Fig. 2a). Mass-corrected MMR also varied amongst individuals and ranged from 0.846 to 6.993 O_2_.min^−1^ kg^−1^ across temperatures (Fig. 2b). At the different test temperatures, MMR ranged between 1.330 and 5.420 O_2_.min^−1^ kg^−1^ at 10°C, between 3.782 and 7.426 O_2_.min^−1^ kg^−1^ at 16°C, between 5.611 and 6.918 O_2_.min^−1^ kg^−1^ at 21°C and between 0.846 and 6.993 O_2_.min^−1^ kg^−1^ at 24°C (Fig. 2b). Aerobic scope ranged from 0.381 to 5.904 O_2_.min^−1^ kg^−1^ amongst individuals, with variation observed in the AS curves (Fig. 2c). At the different test temperatures, AS ranged between 0.563 and 3.969 O_2_.min^−1^ kg^−1^ at 10°C, between 2.907 and 5.864 O_2_.min^−1^ kg^−1^ at 16°C, between 0.990 and 5.904 O_2_.min^−1^ kg^−1^ at 21°C and between 0.381 and 3.746 O_2_.min^−1^ kg^−1^ at 24°C. For all metabolic indices, SMR, MMR and AS, there was a large amount of variation amongst individuals, which was particularly noticeable at the higher test temperatures (Fig. 2).

### Classifying physiological performance phenotypes

Eight individuals were grouped as high performers, 24 individuals were grouped as intermediate performers and 12 individuals were grouped as low performers (Fig. 2). The performance phenotype classification for each individual with SMR, MMR and AS at each test temperature is given in Table A2 of the Appendix. In low performers, SMR across all test temperatures ranged between 0.530 and 3.809 O_2_.min^−1^ kg^−1^ (1.021 ± 0.83; mean ± SD), SMR in the intermediate performers ranged between 0.400 and 3.680 O_2_.min^−1^ kg^−1^ (1.297 ± 0.76; mean ± SD) and in high performers SMR ranged between 0.331 and 3.738 O_2_.min^−1^ kg^−1^ (1.347 ± 0.66; mean ± SD) (Fig. 2). Although there was a significant increase in SMR with temperature (*P*-value = 0.004, *t* = 2.958, *df* = 77; Table 2; Fig. 3a), the relationship between SMR and temperature was not significantly different in the high performers, intermediate performers and low performers (*P* > 0.05, *t* = 0.922, *df* = 77; Table 2, Fig. 3a).

**Table 2:** Linear mixed effects model results for variation in SMR data amongst *C. laticeps* individuals at temperatures of 10, 16, 21 and 24°C. Differences in mass-corrected metabolic rate data between individuals were tested by modelling a second order polynomial relationship between metabolic data and temperature, with a random effects structure weighted by fish ID and temperature. Bold *P-*values depict significant variation in SMR data

**Random effect**	**SD**			
Individual	0.249			
Temperature	0.301			
Residual	0.517			
**Fixed effect**	**Estimate**	**SE**	***t*-value**	***P*-value**
Intercept	1.075	0.530	2.026	**0.046**
Temperature	−0.098	0.070	−1.399	0.166
Temperature^2^	0.006	0.002	2.958	**0.004**
Performance group	0.078	0.085	0.922	0.361
Low performer	1.066	0.537	1.967	0.053
Intermediate performer	0.038	0.137	0.279	0.782
High performer	0.164	0.173	0.950	0.348
AIC	224.70			
Residual SE	0.4721 (*df* = 77)			

--> The MMR in low performers ranged between 1.058 and 6.540 O_2_.min^−1^ kg^−1^ (3.441 ± 1.33; mean ± SD) across all test temperatures. In contrast, in intermediate performers MMR ranged between 0.770 and 6.990 O_2_.min^−1^ kg^−1^ (5.093 ± 2.11; mean ± SD), and in high performers MMR ranged between 3.692 and 7.303 O_2_.min^−1^ kg^−1^ (5.891 ± 1.49; mean ± SD) (Fig. 2). There was a significant positive relationship between MMR and temperature across all test temperatures (*P*-value <0.001, *t* = 4.963, *df* = 77, Table 3, Fig. 3b) and MMR significantly differed by phenotypic group (i.e. high performers, intermediate performers and low performers; *P* < 0.001; *t* = 6.404; *df* = 77; Table 3, Fig. 3b).

Aerobic scope ranged between 0.441 and 3.919 O_2_.min^−1^ kg^−1^ (2.239 ± 0.95; mean ± SD) across all test temperatures in low performers, between 0.381 and 5.904 O_2_.min^−1^ kg^−1^ (3.796 ± 1.68; mean ± SD) in intermediate performers and between 2.738 and 5.822 O_2_.min^−1^ kg^−1^ (4.544 ± 1.17; mean ± SD) in high performers (Fig. 2). There was a significant relationship between AS and temperature (*P-*value = 0.000, *t* = 6.538, *df* = 77; Table 4) and AS significantly differed by performance phenotype (*P* < 0.000; *t* = 7.736; *df* = 77; Table 4, Fig. 3c). High performers exhibited the highest AS across the full range of temperature treatments (Fig. 3c). In contrast, low performers were limited by low aerobic performance in a thermally variable environment (Fig. 3c). Variation in AS was significant at control temperatures of 16°C (*P*-value ≥0.001, *t* = 1.315, *df* = 77) and upper test temperatures of 21 and 24°C (*P*-value = 0.001, *t* = 2.277, *df* = 75), with the variation in the aerobic performance of high, intermediate and low performers being most significant at the high temperature treatments of 21 and 24°C (*P* < 0.05; Table 4; Fig. 3c).

**Table 3:** Linear mixed effects model results for variation in MMR data amongst *C. laticeps* individuals at temperatures of 10, 16, 21 and 24°C. Differences in mass-corrected metabolic rate data between individuals were tested by modelling a second order polynomial relationship between metabolic data and temperature, with a random effects structure weighted by fish ID and temperature. Bold *P-*values depict significant variation in MMR data

**Random effect**	**SD**			
Individual	0.335			
Temperature	1.123			
Residual	1.188			
**Fixed effect**	**Estimate**	**SE**	***t*-value**	***P*-value**
Intercept	−5.019	1.328	−3.779	**0.007**
Temperature	0.881	0.177	4.963	**<0.001**
Temperature^2^	0.021	0.005	4.060	**<0.001**
Performance group	1.138	0.178	6.404	**<0.001**
Low performers	0.915	0.174	5.244	**<0.001**
Intermediate performers	1.645	0.287	4.060	**<0.001**
High performers	2.272	0.361	6.290	**<0.001**
AIC	425			
Residual SE	1.190 (*df* = 77)			

**Table 4:** Linear mixed effects model results for variation in AS data amongst *C. laticeps* individuals at temperatures of 10, 16, 21 and 24°C. Differences in mass-corrected metabolic rate data between individuals were tested by modelling a second order polynomial relationship between metabolic data and temperature, with a random effects structure weighted by fish ID and temperature. Bold *P-*values depict significant variation in AS data

**Random effect**	**SD**			
Individual	0.000			
Temperature	1.181			
Residual	1.004			
**Fixed effect**	**Estimate**	**SE**	***t*-value**	***P*-value**
Intercept	−6.240	1.112	−5.609	**<0.001**
Temperature	0.979	0.150	6.538	**<0.001**
Temperature^2^	0.029	0.004	6.470	**<0.001**
Performance group	1.060	0.137	7.736	**<0.001**
Low performer	1.041	0.146	7.105	**<0.001**
Intermediate performer	1.127	0.220	5.124	**<0.001**
High performer	2.111	0.276	7.648	**<0.001**
AIC	356			
Residual SE	0.859 (*df* = 77)			

## Discussion

An understanding of physiological phenotypic diversity and variation of phenotypes amongst individuals is critical to understand the impacts of thermal variability on fished populations. Using the repeated measures approach of assessing the metabolic response of the same individual at different temperatures, we were able to categorize individuals into physiological phenotypes and to assess phenotypic diversity (i.e. high-performance metabolic phenotypes and low-performance metabolic phenotypes) within the population. Using this approach, we found that the aerobic performance curves of an exploited population of *C. laticeps* were highly heterogeneous. The percentile classification system provided a simple method to identify individuals with a broad aerobic scope (18% of the tested population) that are likely to maintain their performance over the wide range of temperatures characteristic of this increasingly thermally variable coastline.

There were some interesting underlying patterns in the physiology results for *C. laticeps*. Unsurprisingly, temperature influenced SMR, MMR and AS. Standard metabolic rate increased at warmer temperatures. However, there was no significant variation in the SMR amongst individuals at cold temperatures. In contrast to the SMR, there was significant variability in MMR and AS over all test temperatures, although variability did increase with temperature. Environmental variability can reveal differences in the full aerobic scope of individuals, where only those with broad aerobic scope may be able to sustain performance across rapidly changing temperatures.

The variability in aerobic scope phenotypes differed amongst *C. laticeps* individuals by 2-fold in the study, which is in agreement with other studies that found 2- to 3-fold variation in physiological traits between individuals of other fishes (Metcalfe *et al*., 2016; Taboun, 2020; Long *et al*., 2021). This emphasizes the importance of the repeated measures approach for the accurate assessment of phenotypic diversity because not only is the metabolic response to temperature species-specific, but there is significant variability within populations. Although there was 2-fold variation within this sampled population, we were still able to categorize fish, with 18% of the population being high performers, 28% low performers and 54% intermediate performers. The performance phenotypes identified in this study suggest that there is some heterogeneity in physiological performance phenotypes amongst individuals of the exploited Noordhoek *C. laticeps* population.

Categorizing physiological performance phenotypes has implications for assessing the response of fish populations to climate variability. For example, high performers with a broad aerobic scope were able to sustain metabolic performance across changing temperatures, in contrast to energetically limited low performers that experienced reductions in performance outside of optimal temperatures. Future physiological performance phenotypes (i.e. individuals which are likely to be successful in a future environment) will be selected based on their success in the context of future environmental changes. Hence, future performance phenotypes in temperate environments can be predicted by identifying individuals that have a broad range of performance across thermally variable conditions.

It is important to categorize physiological phenotypes not only for understanding the impacts of climate change on populations but also the impacts of exploitation. For example, Duncan *et al*. (2019) compared the thermal physiology of an exploited population of *C. laticeps* from Noordhoek with a protected population from the Tsisikamma National Park (a long-standing marine protected area, where fishing is prohibited) at a population level. They found significant differences in the aerobic scope at a population (not an individual) level, particularly at thermal extremes and attributed this to the selective removal of the physiologically fittest individuals by the fishery. Although the findings in the sampled Noordhoek population support this (i.e. the sampled population was dominated by intermediate and low performers), we were still able to identify high performers in the population. Although exploitation may selectively remove physiological phenotypes from fish populations (Killen *et al*., 2017; Duncan *et al*., 2019), the continued presence of high-performance phenotypes in this heavily exploited population of *C. laticeps* was somewhat surprising. Ultimately the ability of the social group to respond to thermal variability is dependent on the presence of high-performance ‘shoal leaders’ that have the aerobic capacity to cope with thermal variability through phenotypic behavioural responses (e.g. exploratory behaviour and dispersal; Wong and Candolin, 2015; Beever *et al*., 2017; Biro *et al*., 2018).

Regardless of the reasons for the high diversity of physiological phenotypes in this exploited *C. laticeps* population, a comparison of individual variability (using a repeated measures approach) within exploited and unexploited populations is necessary if one would like not only to quantify the impact of exploitation on the thermal physiology of the species, but also better predict the likely response of fishes to thermal change. Generally, fisheries selection of metabolic traits is a research area requiring attention, given that inter-individual variation in metabolic phenotypes will determine differences in how individuals cope with environmental variation (Ward *et al*., 2016).

The percentile method allowed us to identify individuals that exhibited high performance at specific temperatures. Overall, the classification system appeared to yield statistical validity. There was significant variation in the aerobic performance of high, intermediate and low performers at higher temperatures. However, *post hoc* tests indicated that differences in performance were not significant at low temperatures, where several intermediate performers maintained high performance. The performance of some intermediate performers was only high within a narrow range (i.e. either high or low temperatures where performance was higher than the 75th percentile of the mean performance range). In contrast, the physiological performance of these intermediate performers was compromised (i.e. performance was lower than the 25th percentile of mean performance range) at other temperatures outside of this range. These individuals were not high performers across variable thermal environments. Alternatively, some intermediate performers maintained moderate levels of performance across thermal contexts, whereas low performers (i.e. 25th percentile) generally only maintained moderate physiological performance at average temperatures. The percentile classification system could, with some modification, also be suitable for areas that are warming with climate change (rather than experiencing both low and high temperatures), by simply modifying the scoring system (i.e. individuals that performed best at the contemporary and future higher temperatures would receive higher scores).

Individual variability in the shape of performance curves allowed for phenotypic classification and insight into how various phenotypes will respond to changing temperatures. For example, the high-performance individuals (which comprised ~18% of the tested population) that had a broad aerobic scope (i.e. a high AS over a wide range of temperatures) maintained higher than average physiological performance across a thermal gradient (10–24°C) and are likely to be better suited to the increasingly variable thermal environment of the south coast of South Africa. Although it has previously been suggested that generalists may be able to perform over a wider thermal gradient but compromise performance at optimal temperatures (as found in teleost fish by Nati *et al*., 2016), the findings of this study do not support this. Here the high performers had the broad aerobic capacity to remain active and forage across thermal conditions (in agreement with Killen *et al*., 2017, 2021 who reviewed these patterns in shoaling fishes) and will likely be future high-performance physiological phenotypes that will contribute relatively more to the genes of the next generation.

It is possible that the classification did depend on whether fish were tested at upper temperatures of 21°C versus those tested at 24°C and this may be considered a caveat of the study. However, there was no significant difference in the performance between individuals tested at 21°C versus those tested at 24°C. Furthermore, to statistically take into account the varying relationship between metabolic rate and temperature of each individual, temperature was included as a random slope in the random effects structure of the analyses with individual as the random intercept. Hence individuals tested at upper temperatures of 21°C and those tested at 24°C temperatures were both included in this study, owing to the minimal differences in the aerobic performance between the two groups at upper temperatures. In the future, based on the poor performance of *C. laticeps* at the highest test temperature, it is likely that there may be a decline in performance of *C. laticeps* as marine heat waves increase in frequency and intensity (Schlegel *et al*., 2017). High temperatures, even those several degrees below lethal temperatures, not only decrease aerobic performance in stress-compromised low performers, but can cause secondary sub-lethal effects, such as bacterial infections (Lapointe *et al*., 2014; Watson *et al*., 2020,), and damage to heat shock proteins that can result in permanent oxidative damage (Iwama *et al.,* 1999; Pichaud *et al*., 2020).

In conclusion, performance curves can serve as physiological indices that are directly linked to the survival and fitness of fishes. These physiological indices can predict responses to environmental change long before changes to population demographics are evident (the latter are typically used in long-term fisheries datasets; Killen *et al*., 2021). However, mean population physiological parameters (such as AS) may not be useful for predicting how our future populations will respond to a changing climate. The approach used in this study, which included the estimation of individual thermal performance curves data using repeated measures and a percentile scoring system for the classification of individuals according to their physiological phenotype, provided a statistically valid system for categorizing the variability within this fish population. When the predicted changes to the thermal regime were considered, the scoring system could be adapted to estimate the proportion of future physiological performance phenotypes, which will provide insight into the resilience of fish populations to climate change.

## Supplementary Material

Web_Material_coaf043

## Data Availability

All data are incorporated into the article and its online supplementary material.
