# Supplementary material for: Assessing individual physiological variability and future performance phenotypes is essential for predicting the resilience of fish populations to anthropogenic climate change
Source: Conserv Physiol. 2025 Jun 24;13(1):coaf043. doi: 10.1093/conphys/coaf043 (PMC12187193; doi:10.1093/conphys/coaf043)
Supplement: Web_Material_coaf043 [file web_material_coaf043.zip › Appendix.pdf]

Appendix

Table A1: Individual fish weight (mass in kg), length (mm) and sex.

Fish	Mass (kg)	Fork length (mm)	Sex
1	1.453	364	Male
2	0.498	242	Female
3	0.606	270	Female
4	1.49	358	Male
5	0.7	290	Female
6	0.566	253	Intersex
7	1.37	348	Male
8	0.767	292	Female
9	0.592	249	Intersex
10	1.2	329	Male
11	0.692	280	Female
12	1.46	350	Male
13	1.672	370	Male
14	0.846	280	Female
15	0.389	225	Male
16	1.321	338	Male
17	1.151	325	Intersex
18	0.691	270	Female
19	1.561	346	Male
20	1.035	329	Female
21	0.693	260	Female
22	1.414	340	Male
23	1.162	325	Female
24	0.380	215	Female
25	0.821	260	Female
26	1.365	340	Male
27	0.384	230	Female
28	0.443	249	Intersex
29	1.265	-	Male
30	0.534	250	Female
31	0.365	238	Intersex
32	1.456	350	Male
33	1.332	348	Male
34	1.068	323	Intersex
35	0.699	270	Female
36	0.76	275	Female
37	0.338	215	Female
38	0.662	265	Female
39	0.436	308	Female
40	0.48	252	Intersex
41	0.407	216	Female
42	0.642	265	Intersex
43	1.483	340	Male
44	0.685	260	Female

Table A2: Standard metabolic rate, maximum metabolic rate and aerobic scope for each individual at 10 °C, 16 °C, 21 °C and 24 °C. Individuals were grouped into high performers (>75 % percentile) ('HP'), intermediate performers (25 – 75 % percentile) ('IP'), and low performers (<25 %) ('LP') based on the aerobic scope range available for the species.

Fish	Performance category	Temperature (°C)	SMR (O_2.min^{-1}kg^{-1})	MMR (O_2.min^{-1}kg^{-1})	Aerobic scope (O_2.min^{-1}kg^{-1})
1	HP	16	1.0533	6.918	5.864
		10	1.127	4.186	3.059
		21	1.626	6.918	5.292
2	IP	16	0.811	4.915	4.104
		10	0.527	4.156	3.629
		21	1.810	5.611	3.807
3	HP	16	1.681	7.152	5.471
		10	0.830	3.782	2.952
		21	2.299	8.175	5.885
4	HP	16	0.892	6.133	5.241
		10	0.981	3.999	3.018
		21	1.248	6.338	5.090
5	IP	16	0.996	5.105	4.109
		10	0.762	3.959	3.197
		21	2.241	6.620	4.379
6	IP	16	0.830	4.949	4.119
		21	1.439	6.358	4.919
7	LP	16	0.845	3.349	2.503
8	HP	16	1.104	5.220	4.116
		10	0.337	4.267	3.930
		21	1.819	6.710	4.891
9	HP	16	1.152	6.478	5.326
		10	0.851	4.821	3.969
		21	1.853	6.024	4.172
10	IP	16	1.129	5.463	4.334
		10	0.796	3.993	3.197
		21	1.753	6.435	4.681
11	IP	16	1.171	4.363	3.192
		10	0.937	2.597	1.660
		21	1.628	7.532	5.904
12	LP	16	0.906	3.813	2.907
		10	0.774	3.819	3.044
13	IP	16	0.689	3.421	2.732
		10	0.526	2.798	2.272
		21	1.207	7.363	6.163
14	IP	16	1.259	5.395	4.136
		10	0.989	3.247	2.258
		21	3.361	7.644	4.283
15	IP	16	1.027	4.816	3.789
		10	0.523	2.642	2.119
		21	1.870	6.248	4.378
16	IP	16	1.238	5.705	4.466
		10	0.594	3.309	2.714

		21	2.844	7.646	4.802
17	IP	16	1.806	5.413	3.608
		10	1.087	3.716	2.628
		21	2.556	7.020	4.464
18	IP	16	1.278	5.454	4.176
		10	0.767	1.330	0.563
		21	2.251	8.124	5.873
19	LP	16	0.932	2.904	1.972
		10	0.522	2.215	1.693
		21	3.748	6.734	2.986
20	IP	16	0.991	1.981	0.990
		10	0.536	2.160	1.624
		21	2.012	6.082	4.070
21	HP	16	1.211	5.476	4.266
		10	0.714	3.452	2.738
		21	1.490	7.312	5.822
22	IP	16	1.339	5.465	4.127
		10	0.770	2.910	2.140
		21	1.952	8.857	6.901
23	HP	16	1.841	7.426	5.585
		10	1.475	5.420	3.945
		21	3.718	7.971	4.253
24	IP	16	1.146	6.212	5.066
		10	0.598	3.388	2.790
25	IP	16	0.732	3.835	3.103
		10	0.403	3.569	3.167
		21	1.838	6.754	4.916
26	HP	16	1.102	4.719	3.618
		10	0.591	4.116	3.525
		21	1.453	6.723	5.270
27	IP	16	1.695	4.574	2.879
		10	0.831	3.464	2.633
		21	2.410	5.462	3.052
28	IP	16	1.057	4.534	3.478
		10	0.673	2.463	1.789
		21	2.115	7.756	5.641
29	LP	16	1.170	4.918	3.748
		10	0.641	2.130	1.489
		24	2.365	4.387	2.022
30	LP	16	1.470	3.573	2.103
		10	0.688	3.214	2.526
		24	3.532	5.630	2.098
31	IP	16	1.038	4.662	3.624
		10	0.864	2.846	1.982
		24	3.467	6.993	3.526
32	LP	16	0.936	2.976	2.040
		10	0.741	1.997	1.256
		24	1.982	3.712	1.730
33	IP	16	0.939	4.087	3.148
		10	0.714	3.340	2.626
		24	2.219	4.366	2.147

34	IP	16	0.796	5.060	4.264
		10	0.549	3.043	2.494
35	LP	16	0.928	3.433	2.505
		10	0.729	2.469	1.740
		24	2.019	3.651	1.632
36	IP	16	0.879	4.522	3.643
		10	0.698	2.679	1.981
37	IP	16	1.085	2.827	1.742
		10	0.699	2.621	1.922
		24	3.053	6.799	3.746
38	LP	16	0.716	4.295	3.579
		10	0.840	2.261	1.421
		24	0.518	0.959	0.441
39	IP	16	0.909	4.421	3.512
		10	0.705	3.055	2.350
		24	3.019	5.048	2.029
40	LP	16	1.071	4.990	3.919
		10	1.210	2.165	0.955
		24	2.236	3.397	1.161
41	LP	16	0.533	2.616	2.083
		10	1.015	1.9100	0.895
42	LP	16	0.865	3.245	2.380
		10	0.809	2.312	1.503
		24	0.580	1.270	0.690
43	IP	16	0.671	4.113	3.442
		10	0.864	2.621	1.757
		24	0.465	0.846	0.381
44	LP	16	1.038	4.597	3.559
		10	0.843	2.294	1.451

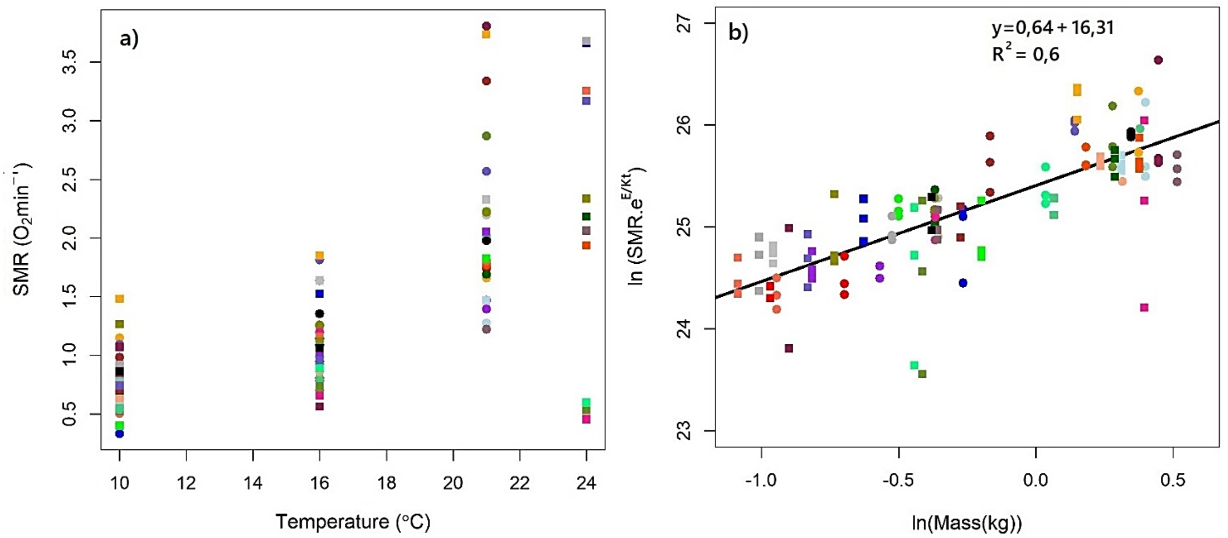

Figure A1: SMR data for an exploited population of *Chrysoblephus laticeps* at temperatures of 10 ˚C, 16 ˚C, 21 ˚C and 24 ˚C. Raw SMR (O2.min^{-1}) data *(a)* was mass-corrected using the linear mass scaling exponent of SMR data, which was determined from the natural logarithm of temperature-corrected SMR data (ln(SMR.e$^{E/kT}$)) plotted against the natural logarithm of mass (ln(Mass(kg)) *(b)*.

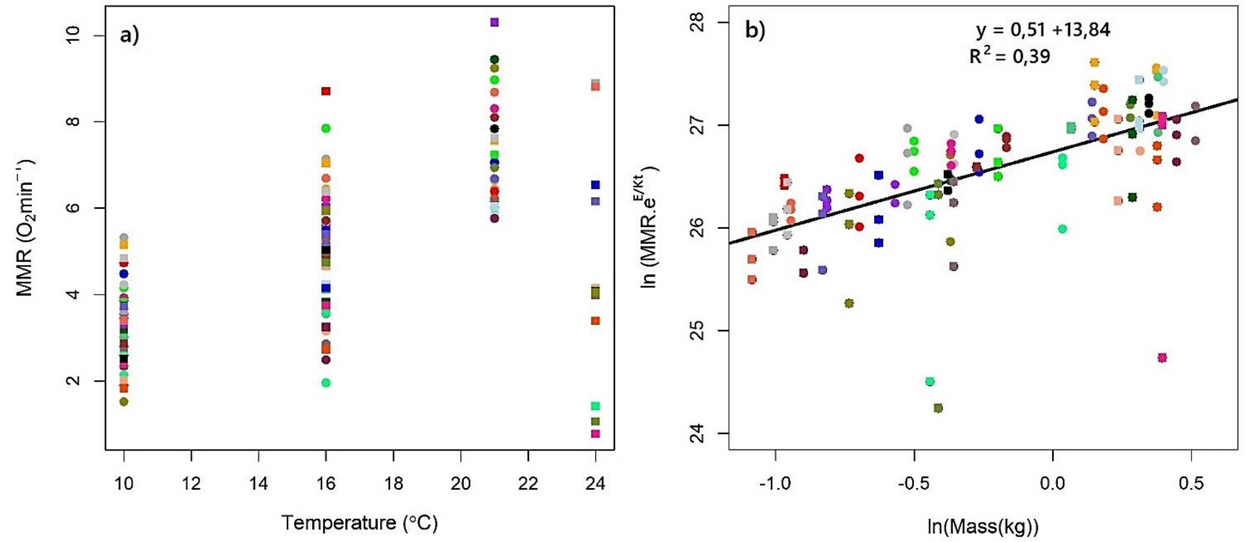

Figure A2: Maximum metabolic rate (MMR) data for an exploited population of *Chrysoblephus laticeps* at temperatures of 10 ˚C, 16 ˚C, 21 ˚C and 24 ˚C. Raw MMR (O2.min^{-1}) data *(a)* was mass corrected using the linear mass scaling exponent of MMR data, which was determined from the natural logarithm of temperature-corrected MMR data (ln(MMR.e$^{E/kT}$)) plotted against the natural logarithm of mass (ln(Mass(kg)) *(b)*.
